# Ciclamilast Ameliorates Adjuvant-Induced Arthritis in a Rat Model

**DOI:** 10.1155/2015/786104

**Published:** 2015-04-27

**Authors:** Zhi-cheng Zhang, Shui-juan Zhang, Bo Jin, Yujin Wu, Xin-fu Yang, Bing Yu, Qiang-min Xie

**Affiliations:** ^1^College of Pharmaceutical Science, Zhejiang Chinese Medical University, No. 548 Binwen Road, Hangzhou 310053, China; ^2^Department of Pharmacology, Zhejiang University School of Medicine, No. 866 Yuhangtang Road, Hangzhou 310058, China; ^3^Laboratory Animal Center of Zhejiang University, Hangzhou 310058, China

## Abstract

We assessed the effect of a novel and selective phosphodiesterase 4 (PDE4) inhibitor, ciclamilast, on chronic inflammation in adjuvant-induced arthritis (AIA), a rat model of rheumatoid arthritis (RA), and acute inflammation in the rat and mouse model of carrageenan-induced paw edema and peritonitis. Our results showed that daily oral administration of ciclamilast at 1, 3, and 10 mg/kg dose-dependently inhibited the increase in hind paw volume of rats with AIA. The inhibition of paw edema was associated with inhibition of both the production of cytokines such as TNF-*α*, IL-1*β*, and IL-6 and cell infiltration assessed in subcutaneous paw tissue. Moreover, there was significantly less tissue destruction in the ciclamilast-treated rats compared to the vehicle-treated rats, as assessed by radiographic analysis and histopathological evaluation. In the two acute inflammation models, ciclamilast inhibited carrageenan-induced paw edema in rats and inflammatory cell migration into the peritoneal cavity in mice in a dose-dependent manner. These results not only suggest that ciclamilast, as a disease-modifying antirheumatic drug (DMARD), can attenuate RA but also provide proof of principle that a PDE4 inhibitor may be useful for the treatment of arthritis.

## 1. Introduction

Rheumatoid arthritis (RA) is a chronic inflammatory autoimmune disease characterized by synovial joints and subsequent progressive erosive destruction of articular cartilage, causing progressive damage to the musculoskeletal system, which contributes to loss of physical function and quality of life [1, 2]. RA prevalence rates in developed populations are approximately 0.5% to 1% of the adult population. The standardized mortality ratios vary from 1.28 to 2.98. Epidemiological studies have consistently demonstrated increased mortality in patients with RA compared with the expected rates in the general population [3]. At present, the drugs used to treat RA range from nonsteroidal anti-inflammatory drugs (NSAIDs) to disease-modifying antirheumatic drugs (DMARDs) such as methotrexate as the main treatment approach, while biological DMARDs such as antitumor necrosis factor (TNF), interleukin (IL)-1, and IL-6 are usually considered only when patients fail to respond to conventional DMARDs. However, the lack of reliable treatment for early RA is a troublesome problem for doctors because most NSAID and DMARD treatments cause severe side effects including stomach ulcers and bleeding in the case of NSAIDs and high blood pressure, osteoporosis, weight gain, and infections in the case of DMARDs [4, 5].

There is therefore a need to develop effective anti-inflammatory drugs with fewer side effects. Phosphodiesterases (PDEs) are a superfamily of enzymes that catalyze the breakdown of cAMP and/or cyclic guanosine monophosphate (GMP) to their inactive forms. PDE4 is the main selective cAMP-metabolizing enzyme in inflammatory and immune cells. Because PDE4 is highly expressed in leukocytes and other inflammatory cells involved in the pathogenesis of inflammatory lung diseases, such as asthma and chronic obstructive pulmonary disease (COPD), inhibition of PDE4 has been predicted to have an anti-inflammatory effect and thus therapeutic efficacy [6]. Targeting PDE4 has enormous clinical potential because it targets a central pathogenic process that bypasses complex antigen receptor-specific immunoregulatory mechanisms. Indeed, selective PDE4 inhibitors have generated substantial interest as treatment for several autoimmune conditions including rheumatoid arthritis, ankylosing spondylitis, Alzheimer's disease, psoriasis, psoriatic arthritis, sarcoidosis, systemic lupus erythematosus, inflammatory bowel disease, atopic dermatitis, and multiple sclerosis [7]. However, early PDE4 inhibitors such as rolipram and piclamilast (RP 73401) have limited and inconsistent efficacy and side effects that make their further development as treatments for these autoimmune diseases less desirable. Therefore, the search for novel structural classes of PDE 4 inhibitors such as apremilast [8, 9] and ciclamilast [10] that may not have the major side effects (nausea, vomiting, and headache) of the archetypal rolipram is ongoing. Ciclamilast is a structural analog of piclamilast. Our previous studies have confirmed that the inhibitory effect of orally administered ciclamilast on airway hyperresponsiveness is due to its inhibition of PDE4 expression, upregulation of cAMP-PDE activity, and downmodulation of PDE4 activity. We have also demonstrated anti-inflammation and antimucus hypersecretion effects in a murine model of asthma [10] and cigarette smoke-induced airway inflammation and injury in mice (unpublished data).

In this study, ciclamilast was carefully evaluated for its immunopharmacological efficacy against adjuvant-induced arthritis (AIA) in rats and its relevant effects on immune responses. We report that ciclamilast strongly inhibited AIA-induced inflammatory responses in rats. Furthermore, it significantly attenuated carrageenan-induced paw edema in rats and carrageenan-induced peritonitis in mice.

## 2. Materials and Methods

### 2.1. Animals

Inbred, female and male, specific pathogen-free ICR mice (22 ± 3 g, 8 weeks old) and Sprague-Dawley (SD) rats (220 ± 20 g, 9-10 weeks old) were purchased from the Shanghai Slac Laboratory Animal Co., Ltd. (China). The animals were housed in a room maintained at 23 ± 2°C with 50 ± 10% humidity and a 12-h light/12-h dark cycle (lights on from 8 : 00 a.m. to 8 : 00 p.m.). The animals were allowed free access to tap water and regular rodent chow. Rodent chow was withheld for 8 h before the final experiments. All of the animal care and handling procedures were approved by the Institutional Animal Care and Use Committee of Zhejiang University.

### 2.2. Adjuvant-Induced Arthritis (AIA) in Rats

Arthritis was induced by inoculation of the rats with Freund's complete adjuvant (CFA). Briefly, on day 0, rats were anesthetized with a mixture of ketamine and xylazine (80 : 10 mg/kg, intraperitoneally) and then injected with 0.1 mL CFA 1 mg/mL of heat-inactivated* Mycobacterium tuberculosis *in 85% paraffin oil and 15% mannide monooleate (Sigma Aldrich, St. Louis, MO, USA) intradermally at the base of the tail. Rats in the control groups were injected with an equal volume of saline instead of CFA. Treatment and group designations are as follows: control (no adjuvant, no treatment); vehicle (2% sodium carboxymethylcellulose (CMC), Sigma Aldrich, St. Louis, MO, USA); 1, 3 and 10 mg/kg ciclamilast (cic, Beijing Joinn Drug Research Center, China); 0.1 mg/kg methotrexate (MTX, Sigma Aldrich, St. Louis, MO, USA), one of the most utilized disease-modifying antirheumatic drugs was used as a positive control and administered by oral gavage. Treatments were given daily from the first injection for a period of 27 days.

### 2.3. Evaluation of Paw Edema in Arthritis

Paw edema was determined by measurement of the paw volume using a water-replacement plethysmometer (YLS-7A; Jinan Yiyan Technology and Science Development Co., LTD, China). Measurements were obtained at baseline (1 day before CFA injection), and day 0 was the first CFA injection. Measurements were also obtained on days 2, 4, 6, 8, 10, 12, 14, 16, 18, 20, 22, 24, 24, 26, and 28 following CFA injection.

### 2.4. Radiographic Analysis and Organ Weights

On day 28, hind limbs were subjected to radiographic analysis using an X-ray machine with a 0.5 mm focal spot, beryllium window, and X-OMAT TL film. The focal film distance was 61 cm, and exposures were 30 s at 45 kVp and 3 mA. Radiographs were analyzed by a board-certified radiologist who was blinded to the treatment groups. Semiquantitative scores were generated for radiographic changes in the joints in the following areas: soft-tissue volume, joint space, subchondral erosion, periostitis, osteolysis, subluxation, and degenerative joint changes. The values were based on increasing severity; day 26 was considered the highest possible score per paw [11]. The spleen and thymus were harvested from each rat, and the wet weight measured to determine spleen and thymic involution, which is the typical of AIA [11].

### 2.5. Histopathology Evaluation

Samples were obtained from the knee joint 28 days after adjuvant injection. The specimens were fixed in buffered 10% formalin and embedded in paraffin. They were serially sectioned onto microscope slides at a thickness of 5 *μ*m and then deparaffinized, stained with hematoxylin and eosin, and evaluated for morphological changes and cellular infiltration. Histopathological changes in the joints due to AIA were described and scored using semiquantitative grading with five scores (0: unremarkable, 1: minimal, 2: mild, 3: moderate, and 4: marked). AIA was scored as follows: 0: normal; 1: minimal synovitis without cartilage/bone erosion; 2: synovitis with some marginal erosion but with joint architecture maintained; 3: severe synovitis and erosion with loss of normal joint architecture.

### 2.6. Determination of Tissue Cytokine Production

To measure tissue cytokine levels, the animals were killed on day 28 by deep ether inhalation, and subcutaneous paw tissues were collected. Samples were placed in PBS containing 0.05% Tween-80, 0.1 mM phenylmethylsulfonyl fluoride, 0.1 mM benzethonium chloride, 10 mM EDTA, and 20 KI aprotinin A, homogenized, and centrifuged at 3000 g for 10 min. The supernatant was rapidly frozen and stored at −76°C for later measurement of IL-1*β*, IL-6, and TNF-*α* levels. Cytokine levels were evaluated using specific rat immunoassay ELISA kits according to the manufacturer's recommendations (eBioscience, San Diego, CA, USA).

### 2.7. Carrageenan-Induced Paw Edema in Rats

Male and female SD rats received a subplantar injection of 100 *μ*L of a 1% (w/v) suspension of *ƛ*-carrageenan (Sigma Aldrich, St. Louis, MO, USA) in the right hind paw [12]. Paw edema was determined by measurement of the paw volume using a water-replacement plethysmometer immediately before subplantar injection of carrageenan and then at 2, 4, 6, and 8 h afterwards. The data are presented as the variation in the paw volume (mL) and were compared to preinjection values. Ciclamilast at 1, 3, 10 mg/kg and 10 mg/kg indomethacin or vehicle (CMC 200 *μ*L) was administered via oral gavage 30 min after intraplantar carrageenan injection.

### 2.8. Carrageenan-Induced Peritonitis in Mice

To study the effect of ciclamilast on carrageenan-induced peritonitis in mice, 1, 3, 10, and 20 mg/mg ciclamilast was administered by oral gavage. Male and female ICR mice received an intraperitoneal injection of 100 *μ*L of a 1% (w/v) suspension of carrageenan. The mice were killed by cervical dislocation under anesthesia 4 h later, and the peritoneal cavity was washed with 1.5 mL heparinized phosphate-buffered saline (PBS) to count peritoneal cells. Total cell counts were performed in a Neubauer chamber, and a differential cell (neutrophils) count of a total of 200 cells was performed using Giemsa staining. The results are presented as the number of total leukocyte cells or neutrophils per milliliter of peritoneal exudate.

### 2.9. Statistical Analysis

Parametric data were evaluated using analysis of variance followed by the one-way ANOVA (Dunnett's method). Nonparametric data were assessed using the Mann-Whitney test. Differences were considered statistically significant at *P* < 0.05. The experiments were repeated at least two times. The SPSS statistical package 15.0 was used for statistical analysis.

## 3. Results

### 3.1. Attenuation of Adjuvant-Induced Arthritis in Rats by Ciclamilast

**Figure 1:**
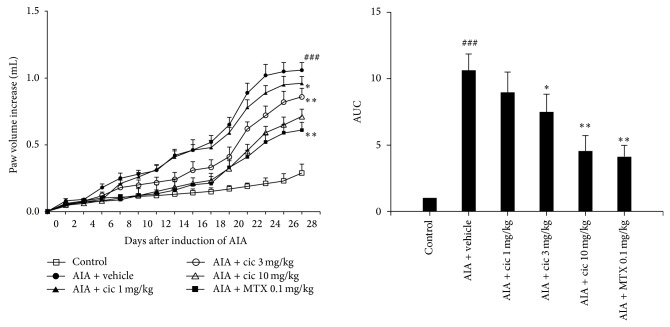
Anti-inflammatory effect of ciclamilast on AIA in rats. Time course of paw swelling on the contralateral paw and the area under curve (AUC) of paw swelling on the contralateral paw in rats with AIA on day 28. Control (no adjuvant, no treatment); vehicle, 1, 3, and 10 mg/kg ciclamilast (cic) and 0.1 mg/kg MTX were administered by oral gavage. Hind paw volume (mL) was measured before and after drug administration using a water-replacement plethysmometer. All drugs were administered by oral gavage. Statistical analysis was performed by one-way ANOVA (Dunnett's method) or Mann-Whitney *t*-test. ^###^ *P* < 0.001 versus control; ^*^ *P* < 0.05, ^**^ *P* < 0.01 versus vehicle. Data represent the mean ± S.E.M. (*n* = 9-10/group).

Paw volume significantly increased in the vehicle-treated rats compared with control rats (*P* < 0.001) from day 8 to day 28. Compared with the vehicle-treated rats, the 3 mg/kg or 10 mg/kg ciclamilast-treated rats and the 0.1 mg/kg MTX-treated rats showed an obvious decrease in paw edema (*P* < 0.05 or 0.01). Similarly, the area under the curve (AUC) of the 3 mg/kg or 10 mg/kg ciclamilast-treated and the 0.1 mg/kg MTX-treated groups also showed a trend toward less paw edema compared with the vehicle-treated rats (each *P* < 0.05 or 0.01) (Figure 1).

### 3.2. Effect of Ciclamilast on Radiographic and Histopathological Changes

**Figure 2:**
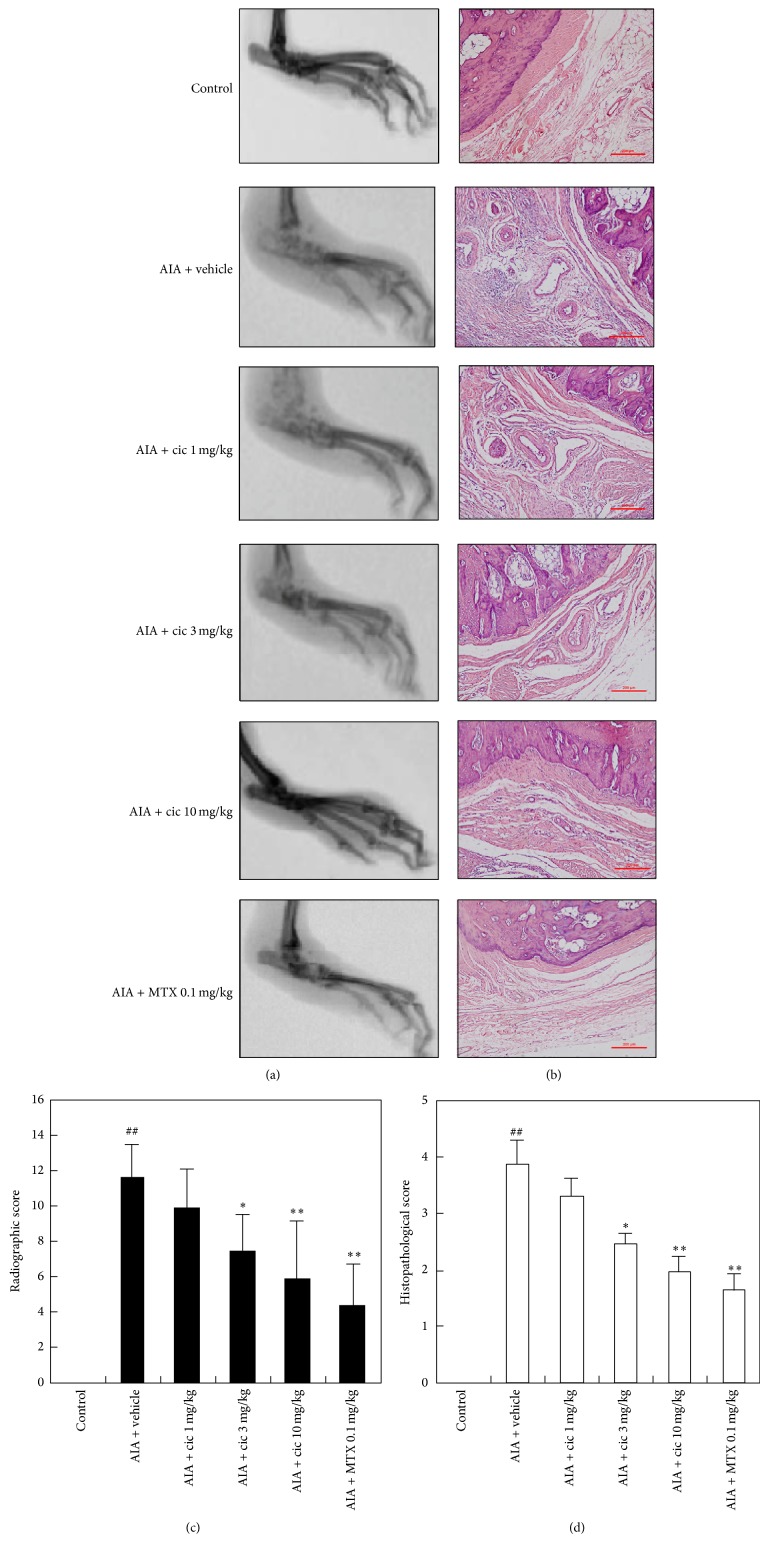
Radiographic (a) and histopathological images (b) of hind paws from representative rats on day 28. Note the evidence of swelling and tissue damage in the treatment rats compared with the control rats (a). Ciclamilast displayed potent and dose-dependent inhibitory effects on both swelling and bone changes and reduced the average radiographic (c) and histopathological scores (d).

As illustrated in the representative day 28 radiographs shown in Figure 2(a), the vehicle-treated rats displayed arthritic changes compared with the control rats. These arthritic changes were characterized by tissue swelling and evidence of bone changes. For example, the vehicle-treated rats had an average radiographic score of 11.6 ± 1.9, whereas the control rats had a mean score of 0 (Figure 2(a)). When administered, ciclamilast displayed potent and dose-dependent inhibitory effects on both swelling and bone changes and showed a trend toward less paw swelling and bone injury when compared with the vehicle-treated rats (each *P* < 0.05 or 0.01). The 0.1 mg/kg MTX-treated group also showed marked improvement compared with the vehicle-treated rats (*P* < 0.01).

To further validate the antiarthritic effects of ciclamilast, the synovial liningand bone erosionswere examined (Figure 2(b)). In the control rats, synovial cells formed a thin layer, and they were flat and quiescent. No leukocyte infiltration or bone erosions were observed. In AIA rats treated with vehicle, the synovial membrane cells became hyperplastic, and it formed a thick, multicelled layer, suggesting active proliferation. In addition, the synovial membrane showed infiltration by leukocytes and hyperanemia with dilated blood microvessels (Figure 2(b)). In the synovial tissues of the ciclamilast- or MTX-treated rats (Figure 2(b)), cell hyperplasia and hypertrophy were significantly inhibited, fewer leukocytes were present, and fewer blood microvessels and bone erosions were seen. The vehicle-treated rats had an average histopathological score of 3.85 ± 0.43, whereas control rats had a mean score of 0 (Figure 2(d)). When administered, ciclamilast and MTX resulted in potent and dose-dependent inhibitory effects on both changes to the synovial lining and bone injury when compared with the vehicle-treated rats (each *P* < 0.05 or 0.01).

### 3.3. Effect of Ciclamilast on Body and Immune Organ Weights

**Figure 3:**
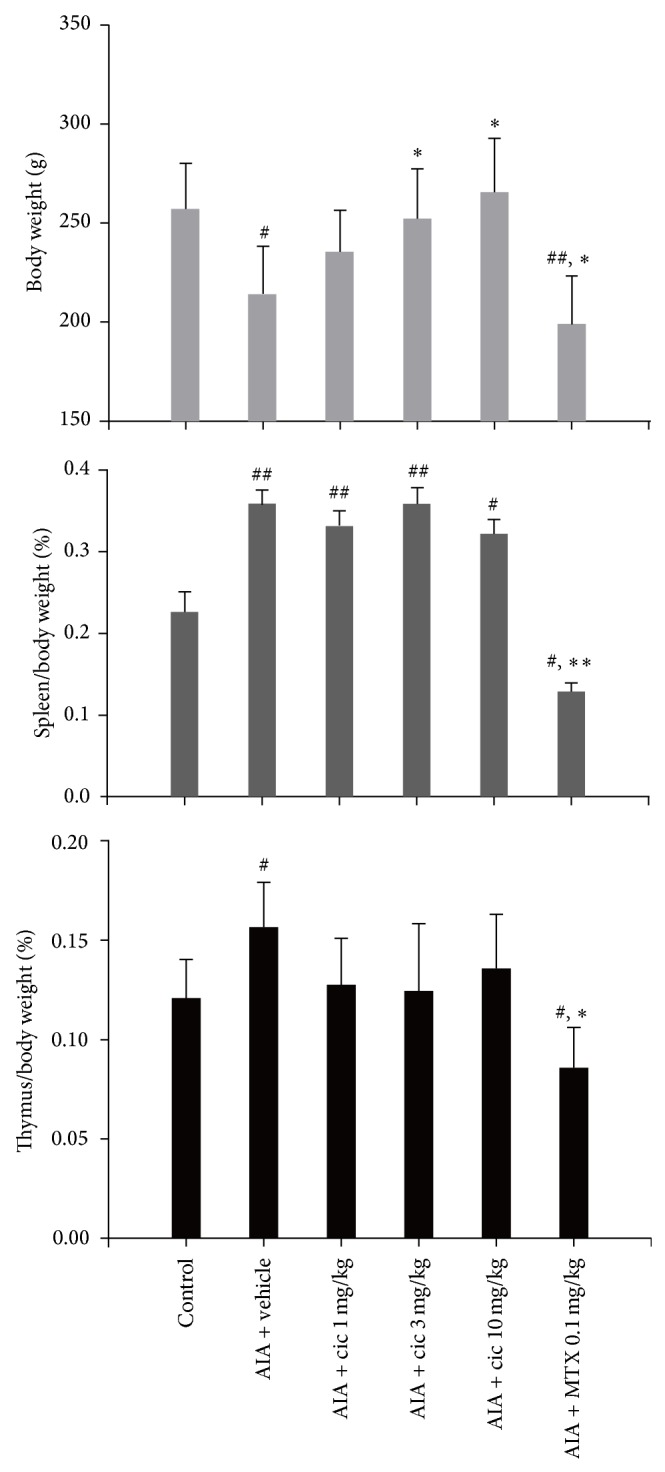
Effect of ciclamilast on body and immune organ weights. The wet weight of organs (spleen and thymus) harvested from rats with adjuvant-induced arthritis on day 28. Statistical analysis was performed by one-way ANOVA (Dunnett's method) or Mann-Whitney *t*-test. ^#^ *P* < 0.05, ^##^ *P* < 0.01 versus control; ^*^ *P* < 0.05, ^**^ *P* < 0.01 versus vehicle rats. Data represent the mean ± S.E.M. (*n* = 9-10/group).

Animals treated with vehicle or MTX weighed substantially less on day 28 compared with the control rats (*P* < 0.05; Figure 3(a)). Treatment with MTX further reduced the rat body weight compared with the vehicle-treated rats (*P* < 0.05). Ciclamilast at 1 mg/kg had no effect on body weight in comparison with the vehicle-treated rats. However, 3 and 10 mg/kg ciclamilast-treated rats exhibited a significant increase in body weight compared with the vehicle-treated rats (*P* < 0.05). AIA rats treated with vehicle or ciclamilast displayed a marked increase in spleen wet weight compared with the control rats (*P* < 0.01; Figure 3(b)). However, the MTX-treated rats had a marked decrease in spleen wet weight compared with the control rats and the vehicle-treated rats (*P* < 0.05; Figure 3(b)). By day 28, the thymus weight in the vehicle-treated rats was markedly increased in comparison with the control rats (*P* < 0.05; Figure 3(c)). Ciclamilast at 1, 3, and 10 mg/kg had no effect on the thymus wet weight compared with the vehicle-treated rats. However, the MTX-treated rats exhibited a marked decrease in thymus wet weight compared with the control rats and the vehicle-treated rats (*P* < 0.05; Figure 3(c)).

### 3.4. Effects of Ciclamilast on the Cytokine Levels of Subcutaneous Paw Tissues

**Figure 4:**
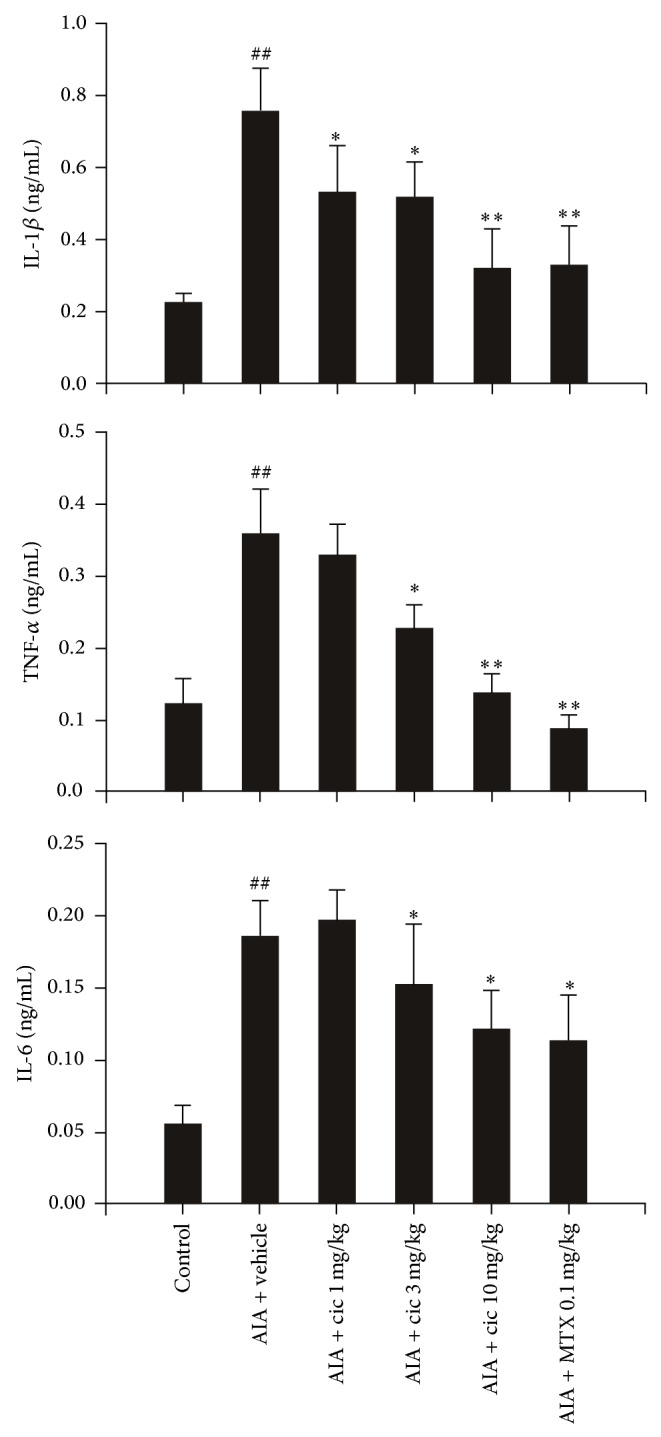
Effect of ciclamilast on the cytokine levels in paw tissue of rats with AIA. Rats with AIA were treated with vehicle or 1, 3, or 10 mg/kg ciclamilast or 0.1 mg/kg MTX via oral gavage from days 0 to 28 after adjuvant induction. Twenty-four hours after the last administration of ciclamilast, rats were killed. The subcutaneous tissue of the right hind paw and the surrounding tarsotibial joints were removed, homogenized, and used for assessment of cytokine levels by ELISA. Statistical analysis was performed by one-way ANOVA (Dunnett's method) or Mann-Whitney *t*-test. ^#^ *P* < 0.05, ^##^ *P* < 0.01 versus control; ^*^ *P* < 0.05, ^**^ *P* < 0.01 versus vehicle rats. Data represent the mean ± S.E.M. (*n* = 9-10/group).

The IL-1, TNF-*α*, and IL-6 levels in paw tissues were significantly increased in vehicle-treated rats compared with control rats (*P* < 0.01) at day 28. Compared with the vehicle-treated rats, the 1, 3, and 10 mg/kg ciclamilast and the 0.1 mg/kg MTX-treated rats showed an obvious dose-dependent decrease in proinflammatory factors (each *P* < 0.05 or 0.01) (Figure 4).

### 3.5. Attenuation of Carrageenan-Induced Edema in Rat Paws by Ciclamilast

**Figure 5:**
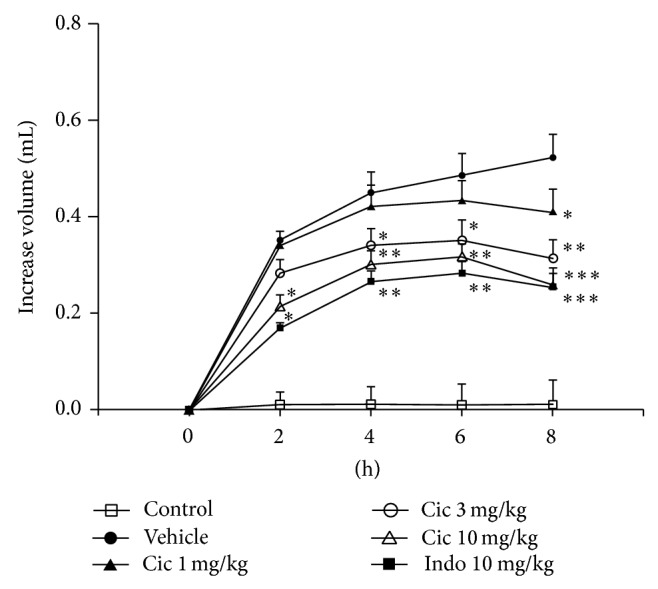
Inhibition of carrageenan-induced paw edema in rats by ciclamilast. Ciclamilast (Cic) or indomethacin (Indo) was administered p.o. 30 min after intraplantar injection of carrageenan (100 *μ*L of 1% carrageenan) into the left hind paw pad. At the specified times, paw volume was measured by a water-replacement plethysmometer. Statistical analysis was performed by one-way ANOVA (Dunnett's method) or Mann-Whitney *t*-test. ^*^ *P* < 0.05, ^**^ *P* < 0.01 and ^***^ *P* < 0.001 versus vehicle group. Data represent the mean ± S.E.M. (*n* = 9-10/group).

Paw volume was significantly increased in vehicle-treated rats compared with the control rats (*P* < 0.001) from hour 2 to hour 8. Compared with the vehicle-treated rats, the 3 mg/kg and 10 mg/kg ciclamilast and the 10 mg/kg indomethacin groups showed an obvious decrease in paw edema (each *P* < 0.05, 0.01 or 0.001). Treatment with 1, 3, and 10 mg/kg ciclamilast inhibited paw edema by 21.4%, 39.9%, and 50.4%, respectively, 8 h after carrageenan administration (Figure 5). The inhibitory effect of 10 mg/kg indomethacin after the same time was 51.1%.

### 3.6. Attenuation of Carrageenan-Induced Peritonitis in Mice by Ciclamilast

**Figure 6:**
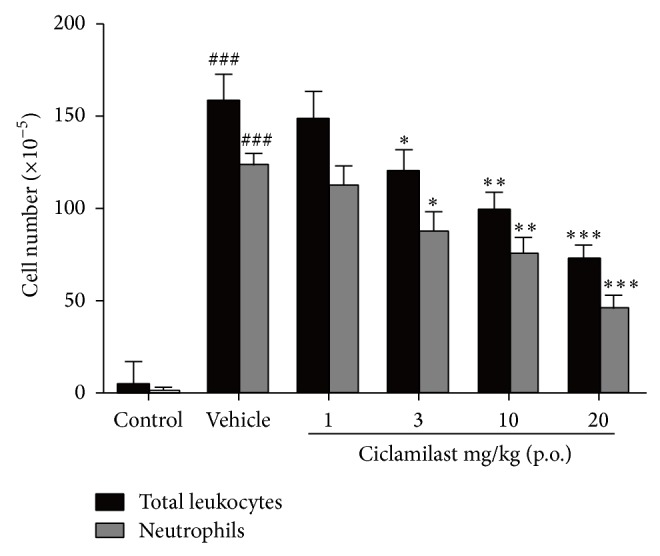
Anti-inflammatory effect of ciclamilast on carrageenan-induced peritonitis in mice. Mice received vehicle or ciclamilast, p.o., followed by injection of 1 mg carrageenan diluted in 100 *μ*L saline solution (i.p.) after 1 h. Mice were killed 4 h later, and the peritoneal cavity was washed with 1.5 mL of heparinized phosphate-buffered saline (PBS) to harvest the peritoneal cells. Statistical analysis was performed by one-way ANOVA (Dunnett's method) or Mann-Whitney *t*-test. ^*^ *P* < 0.05, ^**^ *P* < 0.01 and ^***^ *P* < 0.001 versus vehicle group. Data represent the mean ± S.E.M. (*n* = 8/group).

Figure 6 shows that i.p. carrageenan increased the migration of inflammatory cells into the peritoneal cavity. However, ciclamilast significantly reduced the peritoneal total leukocyte count and neutrophil migration into the peritoneal cavity in a dose-dependent manner. This result was consistent with the fact that neutrophils are the most abundant cells in primary inflammatory exudates.

## 4. Discussion

In the present study, our results showed that daily oral administration of the PDE 4 inhibitor ciclamilast dose-dependently inhibited the increase in the hind paw volume of rats with AIA. The inhibition of paw edema was associated with inhibition of both the production of cytokines such as TNF-*α*, IL-1*β*, and IL-6 and cell infiltration as assessed in the subcutaneous paw tissues. Moreover, there was significantly less tissue destruction as assessed by radiographic analysis and histopathology evaluation in the ciclamilast-treated rats compared to the vehicle-treated rats. These results are in remarkably good agreement with previous studies demonstrating an inhibitory effect of rolipram in other models of arthritis in mice [13, 14]. In addition, Nyman and colleagues clearly demonstrated that the anti-inflammatory effects of rolipram were sustained for at least 7 days after the treatment had ceased [15]. Similarly, Francischi et al. [16] showed that rolipram stopped disease progression for several days in a collagen-induced arthritis model in rats. Overall, these results clearly demonstrate that inhibition of PDE 4 may be of clinical benefit in the treatment of arthritis in humans. In contrast, piclamilast (RP 73401) was not effective in an adjuvant-arthritis model and fluorescein-isothiocyanate- (FITC-) induced ear edema. Cho et al. [17] explained that its ineffectiveness may be due to the weak* in vitro* immunopharmacological properties of RP73401 on nitric oxide (NO) production, TNF-*α* release from differentiated U937, homotypic aggregation of U937 cells, and lymphocyte proliferation triggered by concanavalin A and IL-2. Moreover, it is likely that some pathological inducers such as phorbol 12-myristate 13-acetate (PMA) may alter the pharmacological sensitivity of RP73401 against rheumatoid arthritis-related immunopathological conditions. In the present study, we studied the antiarthritis effects of ciclamilast, a RP73401 structural analog with different side chain groups that was effective against inflammation in AIA compared with RP73401. In addition, ciclamilast also significantly attenuated carrageenan-induced paw edema in rats and carrageenan-induced leukocyte infiltration during peritonitis in mice. We suggest that the effectiveness of ciclamilast in these models may be due to the different side chain groups compared to the RP73401 structural formula. The chemical name of RP73401 is 3-cyclopentyloxy-N-(3,5-dichloro-4-pyridyl)-4-methoxybenzamide, and the molecular formula is C_18_H_18_C_l2_N_2_O_3_. The chemical name of ciclamilast is 2-exonorbornyl-3-cyclopentyloxy-N-(3,5-dichloro-4-pyridyl)-4-methoxybenzamide, and its molecular formula is C_20_H_20_C_l2_N_2_O_3_.

Our previous studies indicated that oral ciclamilast is effective in the treatment of experimentally induced airway inflammation accompanied by reduction of TNF-*α* levels in lung tissues [10]. However, whether ciclamilast can inhibit IL-1*β* and IL-6 and protect against arthritis remained unknown. To confirm our hypothesis of the effects of ciclamilast, we used the AIA rat model. In this model, rats develop chronic swelling in multiple joints accompanied by an influx of inflammatory cells, erosion of joint cartilage, and destruction of joint bone integrity and loss of function. This model of chronic inflammation is due to a complex response involving different proinflammatory cytokines; therefore, there is a possibility of multiple interactions [18, 19]. The AIA model is a well-established experimental model used to study the pathophysiology of various types of human arthritis, particularly rheumatoid arthritis [20, 21]. It is also a good chronic inflammation model for the development of potential anti-inflammatory drugs useful for arthritis treatment [22, 23].

TNF-*α* is the major therapeutic target for rheumatoid arthritis. A key issue in the treatment of chronic arthritis is identifying the crucial molecules driving the transition from the acute phase to the chronic phase of the disease. However, IL-1*β* and IL-6, more than TNF-*α*, appear to be relevant in driving the transition, which suggests that these molecules should be targets for early intervention to stop the progression toward the chronic form of the disease [22]. In the present study, unfortunately, we did not analyze proinflammatory cytokines in the acute phase of the AIA model; we only analyzed the cytokines on day 28. However, we found a significant increase in IL-1*β* and IL-6 as well as TNF-*α* in subcutaneous paw tissues associated with cell infiltration and paw edema. We also found that ciclamilast reduced the TNF-*α* level in addition to the IL-1*β* and IL-6 levels (Figure 4). Our results suggested that ciclamilast may have effects on both the acute and chronic phases of arthritis. To demonstrate the effect of ciclamilast on the acute phase in arthritis, we added two acute inflammatory models, carrageenan-induced paw edema in rats and carrageenan-induced peritonitis in mice. In the two models, ciclamilast inhibited paw edema in rats (Figure 5) and inflammatory cell migration into the peritoneal cavity in mice (Figure 6) in a dose-dependent manner.

In the present study, we also examined joint pathology. The affected hind paws of each animal were removed at the end of the experiment and processed by radiographs and H&E staining. Representative images of radiographs and H&E-stained sections of the proximal interphalangeal joint of the vehicle-treated and ciclamilast-treated rats are shown in Figure 2. The radiographs showed significant bone loss, soft-tissue swelling, periosteal bone formation coupled to a narrowing of the joint spaces between the metatarsals, and decreased bone radiolucency in the vehicle-treated rats. The joint in the ciclamilast-treated rats showed a dose-dependent decrease in tissue swelling, lower periosteal bone formation, less narrowing of the joint spaces, and increased bone density compared with vehicle-treated rats. Radiographic analysis of the ankle joints confirmed destructive joint changes in all AIA groups and the alleviating effects of ciclamilast on joint pathology (Figure 2(a)). Ciclamilast treatment lowered radiographic scores compared with the vehicle-treated rats (Figure 2(c)). The images of H&E-stained sections showed clear flooding of inflammatory cell infiltrate and severe loss of architecture in the joints of the vehicle-treated rats. In contrast, the joint in the ciclamilast-treated rats showed a dose-dependent decrease in inflammatory cell infiltrate in the joint space and damage to the joint architecture with articular cartilage (Figure 2(b)). Ciclamilast treatment lowered the histopathological scores compared with the vehicle-treated rats (Figure 2(d)).

Methotrexate (MTX) is a folate inhibitor; the first reported application in RA was in 1962 [24]. Thereafter, MTX became the most important and most frequently prescribed RA treatment despite several new therapeutic options [25]. The effect of MTX is due to competitive inhibition of folate-dependent enzymes such as dihydrofolate reductase and thymidylate synthase, leading to inhibition of lymphocyte proliferation, and 5-aminoimidazole-4-carboxamide ribonucleotide-transformylase, causing high adenosine levels that in turn have in anti-inflammatory effects [26]. In the present study, we used MTX as the positive control because of this variety of pharmacological actions that are likely to account for its antiproliferative and immunosuppressive effects in rheumatoid arthritis and associated clinical effects [26]. Our results showed that MTX inhibited the increase in hind paw volume of rats with AIA. The inhibition of paw edema was associated with inhibition of both the production of cytokines such as TNF-*α*, IL-1*β*, and IL-6 and cell infiltration as assessed in the subcutaneous paw tissues. Moreover, there was significantly less tissue destruction as assessed by radiographic analysis and histopathology in the MTX-treated rats compared to the vehicle control. The anti-inflammatory effects of the PDE 4 inhibitor ciclamilast in arthritis may be attributed to action on multiple targets, to similar to MTX. However, unlike MTX, ciclamilast is not an immunosuppressive agent and cannot reduce immune organ weights such as the spleen and thymus in rats with AIA (Figure 3). These results are consistent with previous reports of MTX-mediated decreases in the weight of the spleen and thymus [27–29]. In addition, one of the most severe side effects of MTX treatment is the development of hepatic fibrosis [30, 31].

## 5. Conclusion

Using an* in vivo *model of AIA in rats, we demonstrated that treatment with ciclamilast resulted in a potent anti-inflammatory effect and protection against tissue destruction but no immunosuppressive action on immune organs. Mechanistically, ciclamilast inhibited an increase in the expression level of IL-1*β*, IL-6, and TNF-*α*, leading to potential effects on both the acute and chronic phases in the treatment of rheumatoid arthritis.
